# Exploratory Study on Chemosensory Event-Related Potentials in Long COVID-19 and Mild Cognitive Impairment: A Common Pathway?

**DOI:** 10.3390/bioengineering10030376

**Published:** 2023-03-19

**Authors:** Sara Invitto, Paolo Boscolo-Rizzo, Francesco Fantin, Domenico Marco Bonifati, Cosimo de Filippis, Enzo Emanuelli, Daniele Frezza, Federico Giopato, Marcella Caggiula, Andrea Schito, Vincenzo Ciccarese, Giacomo Spinato

**Affiliations:** 1 INSPIRE Lab, Laboratory on Cognitive and Psychophysiological Olfactory Processing, DiSTeBA, University of Salento, 73100 Lecce, Italy; 2 Department of Medical, Surgical and Health Sciences, Section of Otolaryngology, University of Trieste, 34123 Trieste, Italy; 3 Department of Neuroscience DNS, Audiology Unit at Treviso Hospital, University of Padova, 31100 Treviso, Italy; 4 Unit of Neurology, Department of Neuro-Cardio-Vascular, Ca’ Foncello Hospital, 31100 Treviso, Italy; 5 Department of Neuroscience DNS, University of Padova, Audiology and Phoniatrics Unit, Ca’ Foncello Hospital, 31100 Treviso, Italy; 6 Otolaringology Unit, Ca’ Foncello Hospital, Local Health Unit N.2 “Marca Trevigiana”, 31100 Treviso, Italy; 7 Neurology Unit, Vito Fazzi Hospital, 73100 Lecce, Italy; 8 Istituto Santa Chiara, 73100 Lecce, Italy; 9 Department of Neuroscience DNS, Section of Otorhinolaryngology, University of Padova, 35121 Padova, Italy

**Keywords:** OERP, anosmia, Long Covid, MCI, CSERP, EEG, neuro-olfactometry, biomarkers, left frontoparietal network

## Abstract

People affected by the Long COVID-19 (LC) syndrome often show clinical manifestations that are similar to those observed in patients with mild cognitive impairments (MCI), such as olfactory dysfunction (OD), brain fog, and cognitive and attentional diseases. This study aimed to investigate the chemosensory-evoked related potentials (CSERP) in LC and MCI to understand if there is a common pathway for the similarity of symptoms associated with these disorders. Eighteen LC patients (mean age 53; s.d. = 7), 12 patients diagnosed with MCI (mean age 67; s.d. = 6), and 10 healthy control subjects (mean age 66; s.d. = 5, 7) were recruited for this exploratory study. All of them performed a chemosensory event-related potentials (CSERP) task with the administration of trigeminal stimulations (e.g., the odorants cinnamaldehyde and eucalyptus). Study results highlighted that MCI and LC showed reduced N1 amplitude, particularly in the left frontoparietal network, involved in working memory and attentional deficits, and a reduction of P3 latency in LC. This study lays the foundations for evaluating aspects of LC as a process that could trigger long-term functional alterations, and CSERPs could be considered valid biomarkers for assessing the progress of OD and an indicator of other impairments (e.g., attentional and cognitive impairments), as they occur in MCI.

## 1. Introduction

### 1.1. Long Covid and MCI: Is There Overlapping Symptomatology?

Long COVID-19 (LC) is a syndrome characterized by persistent neuropsychological and perceptual symptoms after the first acute episode of COVID-19. This broad spectrum of symptomatology, including olfactory dysfunction (OD), brain fog, and cognitive and attentional diseases, can overlap with that occurring in mild cognitive impairment (MCI) [1]. An increasing number of studies and reports are shedding light on the most frequent consequences experienced by people who went through COVID-19. The U.S. Centers for Disease Control and Prevention estimate that LC symptoms related to mental health may affect one in five adults, regardless of the initial severity of the infection. One study [2] found that, in LC, mild cognitive impairment was still present in 26% of subjects after nine months and that it was associated with factors like older age, male gender, poor education, and a prior history of neuropsychiatric disorders. Another retrospective study on more than a million people showed an increased risk for psychiatric and neurological outcomes such as cognitive deficit and dementia after COVID-19 and that this trend might go on for years, even with weaker variants [3]. Cognitive and olfactory impairments have a strong negative effect on patients’ quality of life, regardless of whether they are caused by a virus or the onset of a neurodegenerative disease. Anyway, MCI and LC could share common or similar pathophysiological, psychophysiological, and neuropsychological mechanisms. COVID-19 attacks the central nervous system (CNS), causing chemo-sensory deficits such as anosmia, encephalitis, cerebrovascular disorders, or brain fog [4,5]. In more detail, the SARS-CoV-2 virus directly invades the CNS due to the interaction between the SARS-CoV-2 spike protein and angiotensin-converting enzyme 2 (ACE-2), which is mostly expressed on neurons in the temporal lobe and hippocampus. Since these brain areas are involved in the pathophysiology of Alzheimer’s disease (AD), SARS-CoV-2 could accelerate the development of neurodegenerative disorders and potentially induce a worsening cognitive decline in MCI and AD patients [6,7,8,9]. Moreover, magnetic resonance imaging performed before and after COVID-19 infection has shown significant changes in experimental subjects’ brain structure compared to controls [10]. In particular, it seems that COVID-19 can cause a reduction in gray matter thickness in the orbitofrontal cortex and the parahippocampal gyrus and increased injury signs in brain areas that are functionally related to the primary olfactory cortex [11].

Infected patients also showed a more significant worsening of cognitive abilities. The studies above provide an anatomical basis for the clinical manifestations occurring after COVID-19 and strengthen the notion of a link between olfactory perception and cognitive decline. The most typical, but not exclusive, symptom of MCI is a fairly slight memory impairment, such as not to interfere with the activities of daily life within a framework of normal general functioning; moreover, both amnesic and non-amnesic MCI can be single-domain, therefore with deterioration in a single cognitive area, or multi-domain [12]. MCI is a heterogeneous clinical category and consequently difficult to identify, since it is placed at an uncertain point between normality and disease and can present sensory, attentional, amnestic, and cognitive symptoms or manifestations that are sometimes difficult to identify. This symptomatology can be similar to the brain fog experienced in LC.

Moreover, recent studies highlighted how MCI subjects have different electrophysiological characteristics and biomarkers [13,14,15,16,17] that identify perceptual and cognitive deficits, and specifically, as for AD and other neurodegenerative processes, also olfactory impairments [17,18]. In fact, olfactory impairment is present in the early stages of MCI, even recalling olfactory impairment as a biomarker of this disease [17,19,20,21,22]. Over time, LC and MCI disorders have found a correlation with neurochemical and neuropathological studies of the olfactory system, with particular implications for neurodegenerative disease. Thus, considering the aspects discussed so far, it is reasonable to ask whether and how Long COVID-19 and MCI can share a common chemosensory pathway.

### 1.2. Olfactory, Psychophysical, and Psychophysiological Assessments

#### 1.2.1. Psychophysical Assessment

To assess and correctly evaluate olfactory perception and value the type and severity of a possible olfactory dysfunction, clinicians can use a combination of objective and subjective tests. Among these, a sensitive and specific self-evaluation procedure carried out by the patient through the use of NHANES (the National Health and Nutrition Examination Survey) [23], a useful tool for the purpose of screening for anosmia that brings together interviews, physical examinations, and laboratory tests, and to combine this self-assessment with an odor identification test (such as the UPSIT or the Sniffin Sticks) [24], recognizing its familiarity and retrieving the name to which to associate it. The identification of the smell will have to take place among different distractions, and it will also be necessary to reduce the cultural and cognitive influences (by presenting, in the latter case, an image that represents the smell) [25].

#### 1.2.2. Psychophysiological Assessment: CSERP as Biomarkers of Olfactory Impairment

Subjects expressing OD undergo multiple visits before turning to a specialist clinic, where they receive preliminary information about their disorder. In short, it is improbable that they enjoy adequate treatment: this could arise, among the various causal factors, from the difficulty in identifying one’s olfactory deficit, and it is precisely for this reason that the patient’s self-assessment must necessarily be accompanied by objective tests, i.e., nasal endoscopy, standardized tests, and instrumental examinations such as magnetic resonance [26], computed tomography [27], electroencephalography (EEG) [18], and chemosensory event-related potentials (CSERP) [16,17,28].

In particular, EEG could detect functional impairment in olfactory perception, highlighting minimal but relevant signal variations [29]. Moreover, detecting olfactory perception is highly complex because olfactory perception has a strong cross-modal component [30,31,32,33].

There are many structures involved in olfactory perception that activate slow and cross-modal components. From an electrophysiological point of view, the olfactory correlated event potentials are called OERPs when the stimulation is a pure, non-trigeminal stimulation. In contrast, they are called CSERPs when the stimulation is of the chemosensory type and activates trigeminal components [34,35,36]. Several chemosensory ERP components have been described [35,36,37,38], such as N1, P3, and LPC. The N1 is an early sensory component and identifies the first-level response to the stimulus; the P3 is a more perceptive and cognitive component that seems to discriminate the stimulus frequency and its hedonic properties and to keep an internal model of the task; and the LPC is a slow positive component that instead identifies the more global perceptual process connected to the recognition and interpretation of the stimulus [35,39]. The CSERP components, if not accompanied by physical stimulations in the EEG (for example, visual or acoustic stimulations, as happened in the first models of olfactory stimulators), are poorly defined components and very difficult to identify because the olfactory stimulus is a slow release stimulus, which also requires a long-term metabolic component, which is the respiratory one [39]. Indeed, the CSERP signal is noisy, according to researchers who usually work on ERP with other modalities [40].

Aside from criticism, various studies identify CSERPs as biomarkers of olfactory deficits or as biomarkers in neurodegenerative disorders, and it is relevant to see how, despite the different techniques used (e.g., classical component analysis [13,15,41], power spectrum analysis [42], or entropic analysis [38], the same type of result was obtained, which confirms component identification as a valid tool to identify functional deficits.

This study aimed to investigate the relationship between LC syndrome and MCI, starting with olfactory EEG biomarkers. EEG signals were recorded from LC, MCI, and control subjects during an olfactory recognition task, and olfactory event-related potentials have been compared across groups. Both MCI and LC showed a reduction in N1 amplitude at the follow-up as compared to healthy subjects. In addition, P3 latencies were slower in LC patients. Such results highlight how the consequences of SARS-CoV-2 can affect the olfactory perception ability at an early stage of smell processing. Furthermore, neurophysiological alterations occur similarly to those observed in MCI patients. The present work encourages the use and study of neuro-olfactory biomarkers, since they provide helpful contributions to intercepting at an early stage those alterations and brain changes directly related to pathological and neurodegenerative conditions.

## 2. Materials and Methods

### 2.1. Subjects

This research involved 18 subjects that had a clinical story of mild COVID-19 disease (mean age 53; s.d. ± 7); 12 patients diagnosed with MCI (mean age 67; s.d. ± 6); and 10 healthy control subjects (mean age 66; s.d. ± 5, 7). The age range of the samples taken into consideration shouldn’t highlight significant differences and variations in sensory and perceptive electrophysiological responses. These differences and variations are present, however, when we compare children with adults or geriatric populations, or young adults with the elderly [43,44,45]. This study was conducted according to the guidelines of the Declaration of Helsinki and was approved by the ethic committees for clinical experimentation in the provinces of Treviso and Belluno (ethic vote: 780/CE), the Friuli Venezia Giulia Region (CEUR-2020-Os-156), and the Ethical Committees for clinical experimentation at Vito Fazzi Hospital, Lecce (Report No. 01–30-01-17).

The LC patients self-reported a persistent (≥3 months) alteration of the sense of smell that began during the acute phase of a RT-PCR-confirmed SARS-CoV-2 infection. Exclusion criteria included a history of previous sinonasal surgery, neurological or psychiatric disorders, and a pre-existing chemosensory dysfunction. All these patients underwent a psychophysical olfactory evaluation using the validated extended Sniffin’ Sticks test battery (Burghart Messtechnik, Holm, Germany), as previously described [46], confirming the OD.

Amnesic MCI patients were recruited after a neurological and neuropsychological assessment according to the latest guidelines and recommendations of the National Institute on Aging and the Alzheimer’s Association (NIA-AA) [12] and of DSM -V [47]. MCI patients showed positive biomarkers for neuronal injury (i.e., hippocampal or medial temporal lobe atrophy and diffuse cortical atrophy on MRI). Moreover, during the recruitment phase, MCI with other forms or causes of dementia and co-morbidity were excluded [48]. The neuropsychological assessment of MCI was conducted through the administration of the Mini-Mental State Examination (MMSE), the Trial Making Test (TMT), the Corsi Test (CT), the Digit Span (DS), and the Rey Auditory Verbal Learning Test (RAVLT).

The healthy subjects (HS) reported neither anosmic nor cognitive symptoms and had not suffered from COVID-19. The HS did not report any current or past psychopathology, neurological illness, or substance abuse and did not report any impairment in normal daily activities.

The LC patients were recruited in the Audiology Unit at Treviso Hospital, in the Department of Neuroscience DNS, Section of Otorhinolaryngology, University of Padova, in the Department of Medical, Surgical, and Health Sciences, Section of Otolaryngology, University of Trieste, and in the Otolaryngology Unit, Ca’ Foncello Hospital, Local Health Unit N.2 “Marca Trevigiana”, Treviso. The MCI patients and the controls were in Neurology Unite and in the INSPIRE lab (DReAM, University of Salento) of Vito Fazzi Hospital (Lecce). The subjects were seated on a chair in a relaxing environment and had to breathe the odorants through a plexiglass tube. The administration of the stimulus was bilateral (in both nostrils). The subject was not asked to perform any task during the stimulation except to breathe and smell the odors presented. The experimental session lasted about an hour.

### 2.2. OERP Recording

EEG signals were recorded using a 16-channel amplifier (Brain Products V-Amp), mounted on an electrode cap equipped with Ag/AgCl electrodes. The Brain Vision Recorder (Brain Products GmbH) software was used for the EEG study. Electrode impedance was kept below 5 kΩ, and the EEG recording sampling rate was 500 Hz. Electrodes were online referenced to FCz and offline re-referenced with a common offline reference over all electrodes. One electrode was placed at the outer canthus of the right eye and used to monitor eye movements. Trials contaminated by eye movements and other artifacts were rejected. The signal was filtered offline (0.01–50 Hz, 24 dB), and the threshold for artifact rejection was set at >|125| μV. Ocular rejection was performed through independent component analysis (ICA). ERP epochs included a 100 ms pre-stimulus baseline correction and a 500 ms post-stimulus segmentation. The averages were calculated for each odorant segmentation. OERP components were labeled N1 and P3 according to Pause et al. [35]. Latency windows were set to 150–300 ms for the N1, and 300–500 ms for the P3.

### 2.3. Olfactometer and Task Methodology

Clinical and healthy subjects have been subjected to an olfactory recognition task with the methodology of chemosensory evoked potentials. As odorants were administered, two predominant trigeminal odors were identified: cinnamaldehyde (CAS number: 14371-10-9) and eucalyptus (CAS number: 470-82-6) [49]. The odorants stimulation had a duration of 1 s, with an interstimulus of 20 sec. Scents were administered via an olfactometer, as in our previous studies [17,28,33,39,41], in two vials, with 20 μL of cinnamaldehyde/eucalyptus provided in 10 mL of Vaseline oil, and both odorous solutions were presented in 20 mL transparent glass vials.

**Figure 1:**
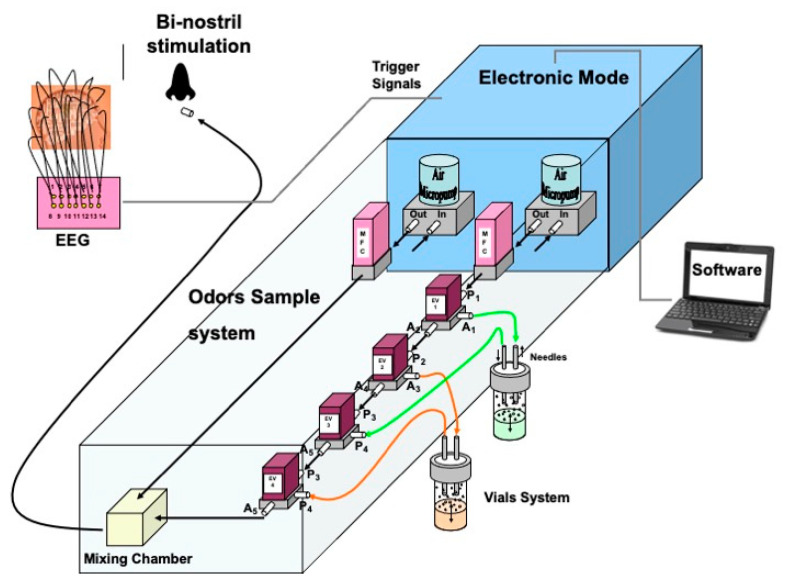
Functional schematic representation of the olfactometer interfaced in EEG.

Both scents were sealed with plastic film and stored in a darkened cabinet. The device used to record odorous stimuli presentation allows the CSERPs evoked by olfactory stimuli to be measured in a controlled, automated fashion, synchronized to the acquisition of the EEG signal (see Figure 1). The trigger points of the stimuli administration in EEG are synchronized with the start of the software embedded in the olfactometer. This method additionally allowed for the blind presentation of smells [4,5]. All the subjects performed the task in the hospitals.

### 2.4. Statistical Analysis:

For the psychometric analysis and processing of the data obtained from the coding of OERP in response to olfactory stimuli, an ANOVA with repeated measures was performed. The group factor (i.e., three levels: MCI, HS, and LC) was considered a between-factor and the electrode factor (i.e., 13 levels: Fp1, Fp2, F3, F4, C3, C4, P7, P8, F7, F8, Cz, Pz, and Fz) as a within-factor. Post hoc analyses were carried out with Bonferroni’s correction.

## 3. Results

### Analysis

Latency: Repeated Measure ANOVA for latency highlighted similar variability in latency between groups in N1 components (see Table 1) (Within Subjects Effect: Electrode F = 0.513; *p* = 0.907; η^2^ = 0.010; Within Subjects Effects: Group F = 0.888; *p* = 0.420; η^2^ = 0.010). P2 latency values showed significant differences in Within and Between Condition (see Table 1); in particular, the Post Hoc Comparison Test (Table 2) highlighted a difference between HS and Long COVID (t = 0.409; P_holm_ = 0.685) and between MCI vs. Long COVID (=3.765; P_holm_ = 0.002). This difference was in favor of the lower values of Long COVID group vs. HS and MCI (HS = 309.55 ms, SD = 112.3; MCI = 339.28, SD = 75; LC = 275,55, SD = 67.7).

**Figure 2:**
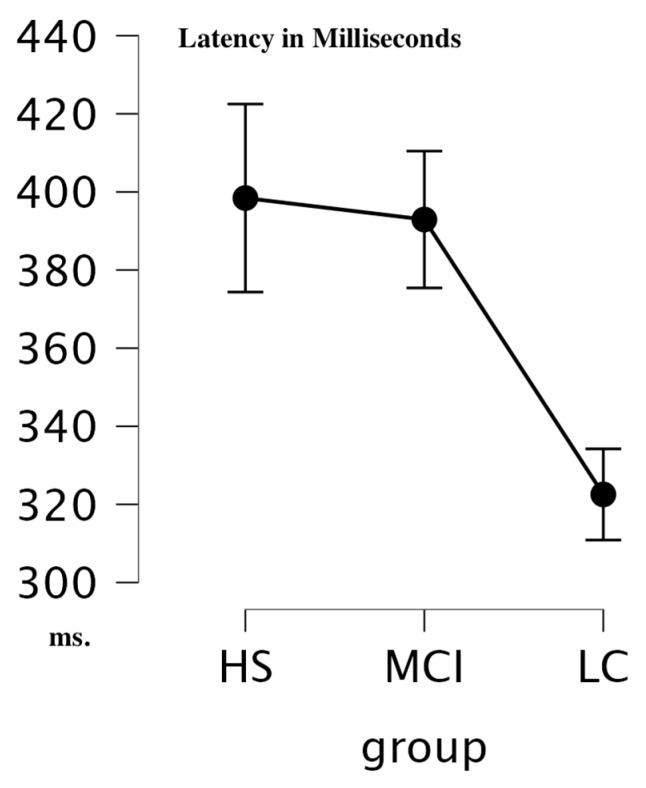
P3 latency comparison in the MS. Long COVID-19 (LC) group showed lower latency values vs. Healthy Subjects (HS) and Mild Cognitive Impairment (MCI). The plot also displays error bars (confidence interval of 95.0%).

**Figure 3:**
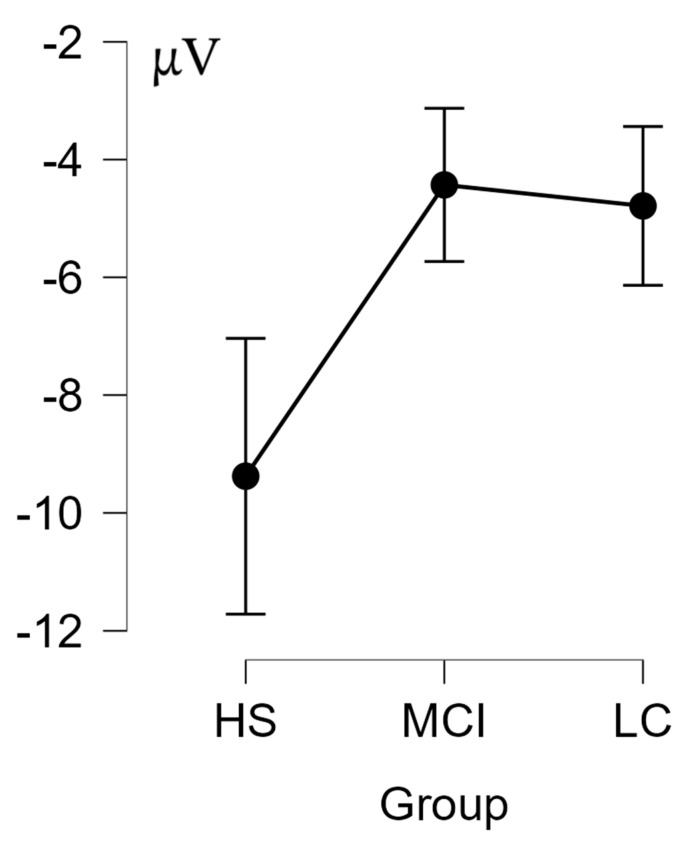
The N1 comparison expressed in μV. Healthy Subjects (HS) group showed a larger N1 component vs. Mild Cognitive Impairment (MCI) and Long COVID-19 (LC). The plot also displays error bars (confidence interval of 95.0%).

**Figure 4:**
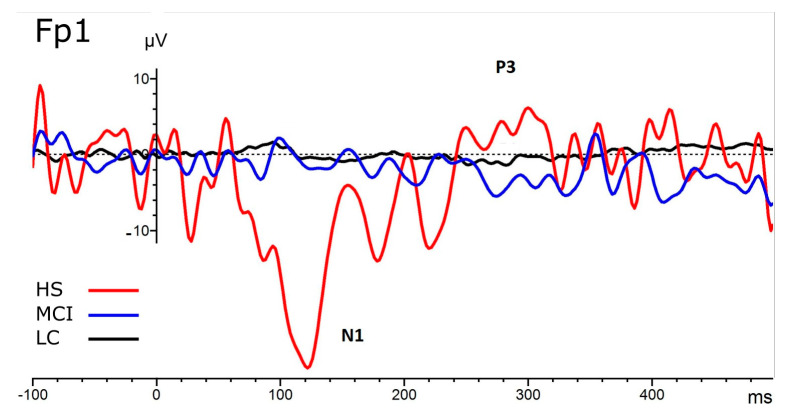
CSERP comparison of averaged signals: red line for Healthy Subjects (HS), blue line for Mild Cognitive Impairment (MCI), black line for Long COVID-19 (LC).

**Figure 5:**
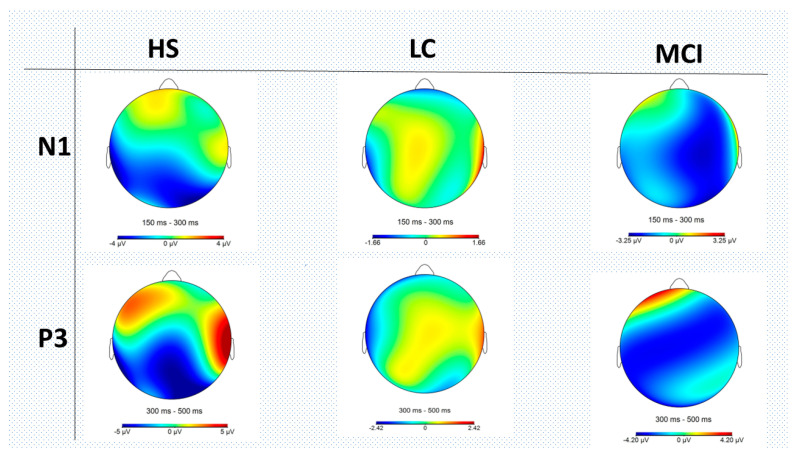
Mapping view of chemosensory event-related potentials (i.e., N1 with a latency range between 150 and 300 ms, and P3 with a latency range between 300 and 500 ms) in Healthy Subjects (HS), Long COVID-19 (LC), and Mild Cognitive Impairment (MCI). The amplitude scale of the Mapping View is different for each group: N1 HS has a value of μV −4 ÷ 4, N1 LC has a value of μV −1.66 ÷ 1.66; N1 MCI has a value of μV −3.25 ÷ 3.25; P3 HS has a value of μV −5 ÷ 5; P3 LC has a value of μV −2.42 ÷ 2.42; and P3 MCI has a value of μV −4.20 ÷ 4.20. This mapping representation shows how cortical activity elicited by chemosensory stimulation in LC is lower than in HC and MCI.

**Amplitude:** Repeated Measure ANOVA for amplitude highlighted different variabilities in amplitudes between groups in the N1 component (see Table 3, Table 4 and Figure 2) (Whitin Subjects Effect: Electrode F = 3.602, *p* = <0.001, η^2^ = 0.063; Electrode*Group: F = 2.172, *p* = 0.001; η^2^ = 0.076; Within Subjects Effects: Group F = 4.170; *p* = 0.023; η^2^ = 0.035). Post hoc analysis of the interaction Electrodes*Group indicated that left frontoparietal positions were more sensitive to these differences, in particular Fp1 (F = 4.891; *p* = 0.014; η^2^ = 0.223) and F7 (F = 3.632, *p* = 0.037; η^2^ = 0.176) in the direction of reduced N1 amplitude for MCI and LC in both positions (see Figure 3, Figure 4, Figure 5 and Table 5).

P3 amplitude values showed significant differences in Within Condition (see Table 3 F = 2.159, *p* = 0.013, η^2^ = 0.045); no interaction was found, and neither effect was found between conditions.

## 4. Discussion

The new coronavirus SARS-CoV-2, responsible for the global COVID-19 pandemic declared by the WHO in March 2020, affects the respiratory tract, usually causing mild and self-limiting symptoms [50]. OD is a frequent symptom during the acute phase of the disease and a predominant persistent symptom in LC [46,51]. It has been reported that the target of the virus may not only be the olfactory neuroepithelium but also the central nervous system, including the central areas that encode the olfactory and gustatory afferents.

Although infection can occur asymptomatically, an estimated 40–45% of the time, the most common and least specific primary symptoms in the prodromal phase include malaise, fever, dry cough, dyspnea, myalgia, and fatigue. However, more severely ill patients develop symptoms such as pneumonia, severe distress syndrome, hypercoagulation, and death [52]. The most serious circumstances are represented by interstitial pneumonia and the spread of the virus to other organs as well. The neurological symptoms, for the most part, manifest themselves early and in the absence of compromise of the respiratory picture; they can be multiple: headache, loss of consciousness, acute cerebrovascular accident, ageusia, anosmia, and damage to skeletal muscles [1,53]. On the other hand, the long periods characterized by stress associated with the infection contribute to the development of long-term neuropsychiatric and neurocognitive symptoms. In fact, it is hypothesized that SARS-CoV-2 causes processes of demyelination or neurodegeneration in the brain [54]. All these alterations have a strong impact on the patient’s life, with more significant relapses in subjects with parosmia rather than hyposmia or anosmia, reporting consequences on a psychological level and high levels of perceived stress, increased by impairments in daily activities [53,55]. For example, the lack of awareness of one’s own smell could generate social phenomena of avoidance and isolation, as well as the moment of the meal could not be lived serenely since the OD negatively affects the appetite and nutritional status of the patient [20]. Going into the merits, OD could be highlighted temporarily or permanently. Underlying etiologic factors include contracting an infection, inflammation of the nasal mucosa, obstruction of the nasal passages, temporal lobe injury or olfactory nerve damage, chronic sinusitis, head trauma, or, in other cases, this dysfunction could represent an early indicator of future neurodegenerative disorders (such as Parkinson’s or Alzheimer’s disease).

Furthermore, patients affected by COVID-19 were identified who, following recovery from respiratory distress, presented long-term OD with an increase, therefore, in the perceptive thresholds of olfactory stimuli [46]. This post-viral OD covers 40% of cases; in fact, the loss of olfactory abilities is certainly one of the symptoms that is most frequently found in affected subjects (about 87% of patients who develop COVID-19 in mild or moderate symptomatology), much higher numbers than the previous coronaviruses, which instead counted a few patients with the same dysfunction [56]. In addition, magnetic resonance studies conducted on affected and anosmic patients have reported changes in the olfactory bulb and in the structures responsible for olfactory perception [11].

In addition to being well defined in terms of OD and attentional/cognitive impairment, the condition connected to LC has not yet been well evaluated with long-term follow-up from an electrophysiological olfactory point of view. The data we present indicate that in LC there is a long-term impairment of the olfactory response. Although there is a slight difference in the age range of the selected groups, the LC group, which is about a decade younger than the controls and MCIs, appears to be the most compromised. This indicates that if age should be a protective factor in sensory impairment in general and chemoreceptive impairment in particular, the LC sample is not protected against this age-related variable [40,43,44,57,58] but results in a relevant sensory impairment, both through evaluation and psychophysical analysis. This impairment is evident in the registered CSERPs, which show a flattening of the early component, the N1 component—a flattening also present in MCI, albeit to a lesser extent. This impairment is evident in the registered CSERPs, which show a flattening of the early component, the N1 component—a flattening also present in MCI, albeit to a lesser extent. The latency in the P3 component, on the other hand, is faster in LC subjects, precisely as a function of the fact that the early sensory component is compromised, so the perceptual/cognitive processing of the chemosensory stimulus does not require significant attentional processing. The wide N1 amplitude herein reported for healthy subjects but not for MCI and LC subjects is in line with previous studies [35] that used chemosensory stimulation (i.e., the administration of smells activating the trigeminal nerve). The shortest latencies of P3 components in LC subjects lead to a more complex interpretation. On the other hand, the position of the impairment seems to be relevant, individuated as the left frontoparietal network, highlighted through the topography of the electrodes as a function of the responses of the samples of subjects analyzed. The left frontoparietal network could be considered a network involved in the control initiation and the ability to rapidly adjust control in response to feedback; it is also involved in altered emotional behavior and attentional and memory impairments [59,60,61]. The involvement of the left frontoparietal network is highlighted in the literature for MCI and is explained as documented by an alteration of memory and cognitive functions [62,63]. In particular, the frontoparietal network is compromised when cognitive decline begins. Finding a common pathway between LC and MCI seems like a relevant starting point, especially if we consider the need for early diagnosis and treatment as well as information on infection prevention. In this case, olfactory or chemosensory stimulations, more than others (e.g., visual or acoustic stimulations), can elicit and highlight an impairment in the left frontoparietal network, which derives from more subcortical structures, as highlighted in the literature [62].

## 5. Conclusions

This study confirms that, after a long-term follow-up of 6 months to 1 year, subjects with LC still present an impairment in the sense of smell. Furthermore, since in LC there are also attentional, emotional, and cognitive symptoms [46,64], so much so that they are associated with the brain fog present in MCI and cancer patients [65], and since all these diagnostic categories can present olfactory biomarkers, the main goal was to compare LC’s CSERP with MCI. From our results, various overlapping cortical patterns emerge, including impairment of the left frontoparietal network [59,61,66].

A reduction of the CSERP components, evident at a topographical level in the left parietal network in the early component, indicates precisely how the deficit starts at a sensory level and then indirectly proceeds to involve other more attentive, emotional, and cognitive aspects.

Starting from this assumption, in addition to a complete psychophysical and psychophysiological evaluation, as we have already carried out previously [11,46], in our future research we will also evaluate, in parallel, the attentional, emotional, and cognitive abilities of these patients. Furthermore, we could compare patients who have had SARS-CoV-2 infection but who are not LC to understand which neuro and psychophysiological characteristics have allowed a more significant impairment in some patients.

The limitations of this study are connected to the fact that the study did not begin with a neuropsychological assessment of these subjects with LC. Furthermore, since the patients had to return to the hospital for a follow-up, there may have been a recruitment bias, in which the patients who accepted to be evaluated after such a long time were the ones who were most affected by the LC symptoms, in particular OD. Therefore, a partially representative sample of LC was self-selected. This could affect the external validity of the study, and, for this reason, we are projecting a robust study on LC, MCI, and HS comparing different odorant stimulation (i.e., trigeminal and pure odorant stimulation) with a full electrophysiological (i.e., auditory ERP, CSERP, and OERP), neuropsychological assessment, and a larger sample size. This new study will allow a comprehensive follow-up of LC and of the connection between LC and the beginning of neurodegenerative processes, in light of the fact that the literature is reporting cases of young people (even adolescents) who, after SARS-CoV-2 infection, have serious neurodegenerative disorders, such as probable Alzheimer disease [67]. This study lays the foundations for evaluating aspects of LC as a process that could trigger long-term functional alterations, and the use of CSERPs could be valid biomarkers for assessing the progress of olfactory, attentional, and cognitive impairments.

## Figures and Tables

**Table 1:** Legend: Repeated Measure ANOVA: Latency of the Chemosensory Event-Related Potentials (CSERP) components N1 and P3. The ANOVA highlights a significant difference in P3 latency between conditions [i.e., group-3 levels: Healthy Subjects (HS), Mild Cognitive Impairment (MCI), and Long COVID-19 patients (LC)].

CSERP	Condition	Cases	F	*p*	η^2^
N1 Latency	Within	Electrode	0.513	0.907	0.010
		Electrode*Group	0.818	0.715	0.032
	Between	Group	0.888	0.420	0.032
P3 Latency	Within	Electrode	2.444	0.004	0.046
		Electrode*Group	0.636	0.910	0.024
	Between	Group	10.119	<0.001	0.074

**Table 2:** Legend: Post Hoc Test Comparison of Chemosensory Event-Related Potential component P3 latency between Healthy Subjects (HS), Mild Cognitive Impairment (MCI), and Long COVID-19 patients (LC).

Group Comparison		Mean Diff	SE	t	P_holm_
HS	MCI	8.309	20.338	0.409	0.685
	LC	72.171	19.434	3.714	0.002
MCI	LC	63.862	16.963	3.765	0.002

Note: *p*-value adjusted for comparing a family of three. Results are averaged over the levels of P3 electrode latency.

**Table 3:** Legend: Repeated Measure ANOVA: N1 and P3 amplitudes. N1 ANOVA highlights significant differences in Within conditions (Electrode and Electrode*Group) and Between conditions (i.e., Group: 3 levels). P3 ANOVA highlights a substantial difference only in the Electrode condition.

OERP	Condition	Cases	F	*p*	η^2^
N1 Amplitude	Within	Electrode	3.602	<0.001	0.063
		Electrode*Group	2.172	0.001	0.076
	Between	Group	4.170	0.023	0.035
P3 Amplitude	Within	Electrode	2.159	0.013	0.045
		Electrode*Group	0.526	0.979	0.022
	Between	Group	2.193	0.125	0.015

**Table 4:** Legend: Post Hoc Comparison of the chemosensory event-related potential (CSERP) component N1 amplitude in Healthy Subjects (HS), Mild Cognitive Impairment (MCI), and Long COVID-19 (LC).

Group Comparison		Mean Diff	SE	t	P_holm_
HS	MCI	−4.947	1.903	−2.599	0.040
HS	LC	−4.590	1.785	−2.572	0.040
MCI	LC	0.357	1.647	0.217	0.830
HS	MCI	−4.947	1.903	−2.599	0.040
	COVID	−4.590	1.785	−2.572	0.040
MCI	COVID	0.357	1.647	0.217	0.830

Note: *p*-value adjusted for comparing a family of three. Results are averaged over the levels of electrodes.

**Table 5:** The N1 amplitude and the mean values (in μV) of the comparison between Healthy Subjects (HS), Mild Cognitive Impairment (MCI), and Long COVID-19 (LC) in Fp1 and F7.

Electrode	Group	Mean μV	SD
Fp1	HS	−20.232	25.64
	MCI	−4.403	3.115
	LC	−3.689	5.488
F7	HS	−10.288	11.55
	MCI	−1.961	3.452
	LC	−4.892	5.821

## Data Availability

Data are available on request to sara.invitto@unisalento.it and francesco.fantin.3@studenti.unipd.it.
